# Women’s Usage Behavior and Perceived Usefulness with Using a Mobile Health Application for Gestational Diabetes Mellitus: Mixed-Methods Study

**DOI:** 10.3390/ijerph18126670

**Published:** 2021-06-21

**Authors:** Shilpa Surendran, Chang Siang Lim, Gerald Choon Huat Koh, Tong Wei Yew, E Shyong Tai, Pin Sym Foong

**Affiliations:** 1 Health Systems and Behavioral Sciences Domain, Saw Swee Hock School of Public Health, National University Singapore, 12 Science Drive 2, Singapore 117549, Singapore; changsiang.lim@u.nus.edu (C.S.L.); ephkohch@nus.edu.sg (G.C.H.K.); mdctes@nus.edu.sg (E.S.T.); pinsym@nus.edu.sg (P.S.F.); 2 Department of Medicine, National University Hospital, 5 Lower Kent Ridge Rd, Singapore 119074, Singapore; tong_wei_yew@nuhs.edu.sg; 3 Department of Medicine, Yong Loo Lin School of Medicine, National University of Singapore, 10 Medical Drive, Singapore 117597, Singapore

**Keywords:** diabetes, gestational, follow-up studies, mentoring, mobile applications, telemedicine, human, pregnancy, female

## Abstract

The prevalence of gestational diabetes mellitus (GDM) is increasing, and only a few mobile health (mHealth) applications are specifically designed to manage GDM. In this mixed-methods study, a follow-up study of a randomized controlled trial (RCT) analyzed a largely automated mHealth application-based lifestyle coaching program to (a) measure the application’s usage behavior and (b) explore users’ perceptions of its usefulness in GDM management. Quantitative data were collected from the 170 application users who had participated in the intervention arm of the RCT. Semi-structured interviews (n = 14) captured users’ experiences when using the application. Data were collected from June 2019 to January 2020. Quantitative data were analyzed descriptively, and interviews were analyzed thematically. Only 57/170 users (34%) logged at least one meal, and only 35 meals on average were logged for eight weeks because of the incorrectly worded food items and limited food database. On the contrary, an average of 1.85 (SD = 1.60) weight values were logged per week since the weight tracking component was easy to use. Many users (6/14 (43%)) mentioned that the automatic coach messages created an immediate sense of self-awareness in food choices and motivated behavior. The findings suggest that for GDM management, a largely automated mHealth application has the potential to promote self-awareness of healthy lifestyle choices, reducing the need for intensive human resources. Additionally, several gaps in the application’s design were identified which need to be addressed in future works.

## 1. Introduction

Gestational diabetes mellitus (GDM) is a well-established risk factor for future type 2 diabetes mellitus [1]. GDM affects 20–30% of pregnancies in Singapore, which is way above the global prevalence of 13.8% [2]. In Singapore, the current care plan for women with GDM includes providing them with information on health, nutrition, and self-care through face-to-face consultations [3]. These activities are resource-intensive, do not allow for learning to be spaced over time, and lastly, do not let patients revisit the information at their preferred time and pace [3]. Moreover, since patient support occurs only during face-to-face consultations, and consultations are typically short and a few months apart, information may not be delivered on time [3]. Hence, there is a need to explore alternate ways to provide health information to women with GDM. 

Mobile health (mHealth) applications have been shown to be an effective alternative to face-to-face consultations for delivery of health-related information because the information can be delivered promptly. Additionally, mHealth applications have been shown to improve patients’ compliance with treatment and their self-management awareness [4,5,6]. Many mHealth program types are already established in Singapore according to the 2015 World Health Organization’s Third Global Survey on eHealth [7], and Singapore has one of the highest mobile phone penetrations (154% in May 2019) in the world [8]. Singapore is in the best position to develop mHealth applications to manage GDM because of the increased smartphone penetration and high prevalence of GDM. Recent studies from Singapore have shown that mHealth usage for disease monitoring ranges from 20% to 33% [9,10]. Despite this, we believe that mothers with GDM are highly likely to be receptive to mHealth application usage in GDM [1]. Firstly, women in the reproductive age group are younger and generally tech-savvy [11]. Secondly, there is less likelihood of technology fatigue since GDM is time-bound [12]. Lastly, pregnant women are a motivated group willing to engage with health monitoring due to the risk of perinatal complications [11,12]. 

Globally, only a few mHealth applications are designed to support self-management of GDM [13,14,15,16,17]. Most of these mHealth applications are resource-intensive, involving large components of proactive manual feedback by the healthcare team [3]. For example, some applications [13,14] applied a two-way in-application correspondence between the healthcare professionals and the users. The healthcare professionals would provide individualized daily manual feedback and promptly answer questions raised via the application. Similarly, using other applications [14,15,16], the healthcare team would proactively reach out to the users upon receiving alerts when the glucose levels were abnormal.

On the other hand, studies on the effectiveness of largely automated mHealth applications in managing GDM are limited, i.e., an mHealth application consisting of an interactive coaching system with minimal manual intervention from healthcare professionals. One such study is the SMART-GDM study [3]. This study has shown clinical benefits when using a largely automated lifestyle coaching program for GDM management [3]. Our objective is to evaluate such an automated and resource-lean mHealth application for GDM in Singapore because it is also important to understand the user experience for applying the application in a clinical setting. Since mHealth interventions are complex, involving multiple components with different behavioral constructs, it is hard to predict which component will have the maximum impact [18,19]. However, qualitative research methods can help us to understand the application’s implementation process, how well the application is received by the target user group, and its unanticipated effects, if any [18,19]. Such studies will help GDM application developers in the optimization of the mHealth application for the target population as well as identification of its most active component. Therefore, to ensure the mHealth application’s effective incorporation in a clinical setting, this mixed-methods study investigated two objectives: (a) to measure the application’s usage behavior and (b) to explore how users perceived the application’s usefulness when managing GDM. We used a mixed-methods study design because by combining both quantitative and qualitative results, it helps us to obtain a deeper understanding of the users’ perspectives without over-reliance on a particular method [20]. 

## 2. Materials and Methods

### 2.1. Habits-GDM Application Overview

The mHealth application, i.e., Habits-GDM, analyzed in this study is a largely automated mHealth application-based lifestyle coaching program minimizing the need for intensive human resources. 

**Figure 1:**
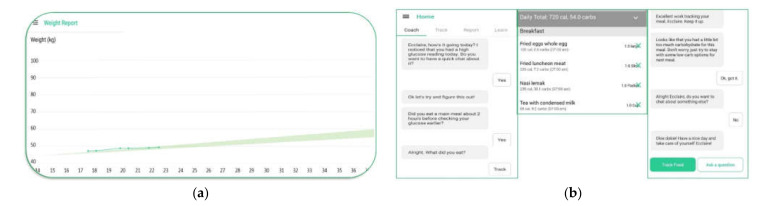
Habits-GDM application user interface. (**a**) Screenshot of weight tracking, and (**b**) screenshot of automated messages sent post-entry of glucose readings.

Habits-GDM has several components built in to increase women’s self-awareness when managing GDM. The application has three key components: interactive educational lessons, tracking tools (self-monitoring blood glucose, physical activity, diet, and weight), and coaching [3]. Table 1 describes the components of the Habits-GDM application. Figure 1 shows screenshots illustrating the Habits-GDM application’s user interface. Supplementary Material Figures S1 and S2 show screenshots of the educational lessons and various tracking interfaces on Habits-GDM application, respectively.

### 2.2. Theory Used in the Design of the Habits-GDM Application

Health Belief Model constructs was used to develop the components of the Habits-GDM application [3,23]. The Health Belief Model was used because it is one of the widely used theories applied to mHealth interventions for behavior change and it is considered relevant for pregnant women [24,25]. The constructs of the Health Belief Model are perceived susceptibility, perceived severity, perceived benefits, perceived barriers, self-efficacy, and cues to action [23]. Perceived susceptibility is described as a person’s perception of his own vulnerability to health threats [26]. Perceived severity is defined as a person’s assessment of the level of health threat, i.e., seriousness of the health threat [26]. A person decides to take preventive action based on the combined effect of perceived susceptibility and severity [26]. Perceived benefit refers to the usefulness of adopting a behavior and it will likely affect the course of action [26]. Perceived barrier refers to a person’s perception of how much effort he needs to make to change the behavior. For example, if an mHealth application is easy to use, the more useful it can be [27]. Perceived self-efficacy reflects a person’s confidence in his/her capabilities to successfully perform a behavior [26]. It thereby affects the person’s choice, effort, and persistence related to this behavior [26]. Cues to action is defined as an external reminder to continue the preventive action so that a person’s need for remembering can be reduced [26,27]. The combined effects of self-efficacy and cues to action provide the push to preventive action. 

### 2.3. Study Design

This study was a follow-up study of the SMART-GDM randomized controlled trial conducted at the National University Hospital, Singapore, which sought to examine the effectiveness of the Habits-GDM application designed for women with GDM (Clinical trial reg. no. NCT03249896, clinicaltrials.gov accessed on 20 June 2021). The SMART-GDM study recruited a total of 340 women with GDM (170 women in the intervention arm and 170 women in the control arm). The SMART-GDM aimed to determine whether the Habits-GDM application could prevent excessive gestational weight gain and improve glycemic control and maternal and neonatal outcomes in GDM [3]. Usual care for women with GDM at the National University Hospital includes one group-based face-to-face education session jointly conducted by the diabetes nurse educator and the dietician. This session is conducted around one to two weeks after the diagnosis of GDM. The sessions cover how to monitor blood glucose and instructions on diet and lifestyle modifications. 

**Figure 2:**
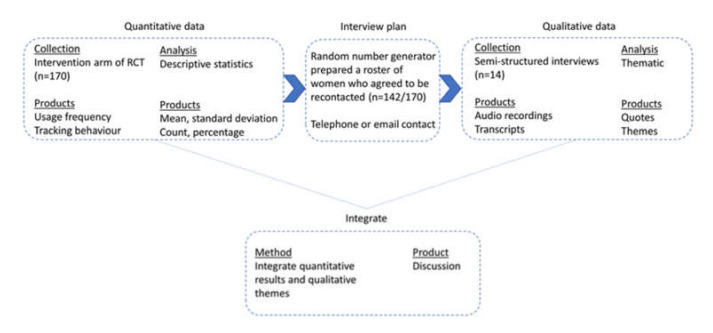
Explanatory sequential mixed-methods design. RCT—randomized controlled trial.

The current study used an explanatory sequential mixed-methods study design. As Figure 2 shows, the study was conducted in two phases, using quantitative components in phase 1 and qualitative components in phase 2.

#### 2.3.1. Sampling and Data Collection—Quantitative Data

Quantitative data of the application’s usage behavior were collected between June to July 2019 from the 170 Habits-GDM application users who participated in the intervention arm of the SMART-GDM. The application collected information on users’ application usage frequency, i.e., frequency of educational lessons accessed and coaching messages received. This data was collected using Google Analytics Service [28] (Figure 2). Additionally, the application also collected information on users’ weight and diet tracking behavior. Jana Care, Inc Singapore. [29] kept the de-identified data. 

#### 2.3.2. Sampling and Data Collection—Qualitative Data

For the qualitative component, we interviewed women from the intervention arm of the SMART-GDM who had previously consented to be recontacted for related studies: 142/170 women. Using a random number generator, we prepared a roster of these women. They were sorted in rank order and contacted in sequential order by a research coordinator from November 2019 to January 2020 via telephone or email, stating the objectives of the study. Nine women declined to participate due to a busy work schedule, and we reached data saturation by the 14th interview. Data saturation was decided based on reaching thematic saturation, where we could no longer identify new themes from the interview data [30]. The semi-structured interview guide (Supplementary Material 2) was developed based on the findings from the quantitative analysis of the application’s usage data and published literature [31,32]. Interview domains included experiences of using the educational lessons, tracking tools, and coaching, and perceptions of its usefulness when managing GDM. After obtaining written informed consent, all interviews were audio-recorded and conducted in English by two authors. Each interview lasted for about 60 min. We conducted the interviews at the National University Hospital and reimbursed the women with an SGD 30 voucher for their time.

### 2.4. Ethical Considerations 

The National Healthcare Group Domain Specific Review Board reviewed and approved this study (Reference code: 2019/00666). We obtained written informed consent from the study participants.

### 2.5. Data Management and Security

We maintained confidentiality by sharing only de-identified data with Jana Care. All research materials are stored separately in a standalone password-protected desktop at the National University Hospital, accessible only to the research team members. Hardcopy research data is kept under lock and key at the principal investigator’s office, which requires an access code for entry and is accessible only to the research team members.

Jana Care is a global company that specializes in building smartphone applications to improve the health outcomes of people living with chronic diseases [29]. It was founded in 2011 and is registered with the United States Security and Exchange Commission (SEC CIK #0001696563) [33]. The Habits-GDM application and its web platform follow the data privacy and security policies mandated for Health Insurance Portability and Accountability Act compliance. Jana Care conducts regular audits of their information technology system to maintain compliance. The Habits-GDM application can be accessed by participants only using a passcode, username, and password. In an event whereby the participant’s smartphone is either lost or stolen, the research team can remotely wipe the data through the Habits-GDM administrator portal.

#### 2.5.1. Data Analysis—Quantitative Data

Quantitative data was analyzed from August to November 2019 and presented as mean and standard deviation (mean (SD)) or as count and percentages (n (%)) for continuous variables and categorical variables, respectively. Demographic characteristics and health information details of the participants of the intervention arm of the SMART-GDM and the qualitative interviews were analyzed using R statistical software (version 3.6.1, R Foundation for Statistical Computing, Vienna, Austria). The qualitative interview participants are a subset of the participants from the intervention arm of the SMART-GDM. One author read, manually categorized, and tagged the coach messages with a categorical label. 

#### 2.5.2. Data Analysis—Qualitative Data

Interviews were transcribed verbatim and analyzed thematically based on a thematic analysis framework [30] from February to April 2020. After verifying the accuracy of the transcripts with the audio recordings, two authors independently re-read two transcripts multiple times for familiarization. We used a three-stage coding process, i.e., open, axial, and selective coding, to analyze the data using QSR NVivo 12 software [34,35,36]. In the first stage, i.e., open coding, two authors examined the data line-by-line [36,37]. For instance, the data ‘I found the educational lessons useful because the content was represented in pictures, and it was of short duration’ was broken down into ‘pictorial representation’ and ‘short duration’. In the second stage, i.e., axial coding, we systematically grouped the broken data by developing connections among the categories. The categories were then identified to develop axes [36,38]. For instance, ‘difficult search feature’ and ‘limited food database’ were grouped as ‘reasons why less useful’ which was connected to the phenomenon for behavior change in GDM management. In the third stage, i.e., selective coding, the qualitative data was assessed to identify the main phenomenon of the study [36,38]. In this way, we developed a preliminary codebook. We then applied the preliminary codebook to another randomly selected transcript to determine whether most of the codable units fit within this codebook or whether new codes needed to be added. After this iterative process, the two authors developed a final codebook, and one author applied it to the remaining transcripts. Using a constant comparative method, we compared new data with existing data and codes, and the final codebook was again refined so that it was representative of the data. To improve the rigor of the analytical process, firstly, we asked the third co-author of this study to look through the derived codes and themes. Secondly, doubts regarding the codes and themes were clarified and resolved through regular team meetings. The final codebook included four thematic sections. Thematic saturation was reached when no new codes and themes emerged from the data. Each quote includes P, followed by a participant number (P01). 

We enhanced the study’s trustworthiness by adopting several measures throughout the data collection and analysis phase [39]. Specifically, the interviewers acknowledged their position as a member of the research team with the participants. This strategy helped to mitigate any pre-conceived bias arising due to the interviewers’ role in the research team. Additionally, the interviewers wrote memos after each interview to document their reflections and any emerging themes. The quantitative data of the qualitative data are reported as counts (n) according to Neale et al.’s guidelines [40]. 

### 2.6. Integrating Quantitative and Qualitative Data

We used O’Cathain, Murphy, and Nicholl’s (2010) guidelines [41] to integrate the quantitative results with the qualitative findings by following a thread. This technique is best suited to the current study’s sequential design. We analyzed the phase 1 quantitative data and phase 2 qualitative data separately. Themes or threads from one phase were followed through the other phase so that the quantitative and qualitative findings in the same theme could be integrated and interpreted together.

## 3. Results

See Supplementary Material Table S1 for the demographic characteristics and health information details of the participants of the intervention arm of the SMART-GDM and the qualitative interview. In brief, the mean age of the participants of both groups was 32 years. Majority of the interview participants were ethnically Chinese, compared to those in the intervention arm of the SMART-GDM (57% vs. 44%, respectively). The mean number of weeks of gestation at diagnosis and delivery was 25 and 39 weeks respectively, for both the groups. The interview participants were on average 17 (SD = 5.63) months postpartum when participating in this study. The themes and subthemes developed after integrating the quantitative and qualitative findings are summarized in Table 2 and described below.

### 3.1. Themes

#### 3.1.1. Use of Educational Lessons of Habits-GDM Application

Only half of the application users (84/170, 49%) accessed at least one educational lesson. Of the 84 users, more than half (56/84, 67%) accessed the ‘Glucose monitoring’ and ‘Healthy eating’ lessons. ‘Why exercise’ was the least frequently accessed lesson (46/787, 6%). See Supplementary Material Table S2 for the usage frequency of the educational lessons. 

##### Reasons Why Educational Lessons Were Useful and Less Useful

Most of the qualitative interview participants (9/14) mentioned that the educational lessons helped them manage their GDM because of the short duration (6/9), pictorial representation (6/9), and easy to understand content (6/9). They also said that having the educational lessons was an advantage because all the necessary health-related information was available in one place (3/9). However, a few others (5/14) did not find the educational lessons useful to manage GDM because of the basic (simple) content (3/5), and already available information on websites (2/5). Despite identifying their benefits, participants mentioned that a mHealth application could not replace a healthcare professional since their medical needs were unique. However, they suggested increasing telemedicine coaching to reduce the frequency of hospital visits:

*“The application information was basic. Whereas the information provided by the healthcare professional is detailed […] dietician had all the products on her shelf […] nurses even bought a bowl and showed us the amount that we could take […] application cannot replace them […]” (P14)*.

##### Reasons How Educational Lessons Were Useful and Less Useful

The qualitative interview participants (7/14) named ‘Healthy eating’ as the most useful lesson because it helped them to remember the healthy foods (7/7) and guided them to make healthy food choices (7/7) when managing GDM. ‘Eating smart’ and ‘Eating out’ were the next most useful lessons. The quantitative data showed that the ‘Glucose monitoring’ lesson was most frequently accessed (127/787, 16%). It could likely be due to the main focus on blood glucose monitoring in GDM. Consistent with the quantitative data, the interview participants (9/14) named ‘Why exercise’ as the least useful lesson. They mentioned feeling tired due to pregnancy and hence did not exercise. Five out of the fourteen interview participants did not mention any lesson as the least useful when asked. 

#### 3.1.2. Diet Tracking Behavior with Habits-GDM Application

Users were prompted to log at least three meals in the application for any two days of a week (6 meals/week). However, among the 170 application users, only 0.88 (SD = 0.99) number of meals were logged per week on average, with only one user (0.6%) logging six or more meals. Among the 170 application users, 113 users (113/170, 66%) did not log any meals using the application. Only 57 users (57/170, 34%) logged at least one meal using the application and, on an average, 35 meals were logged by them for 8–10 weeks. The number of meals logged represents those meals logged using the application and do not include entries on the paper diary, which is part of usual care. 

##### Reasons Why Diet Tracking Component Was Less Useful

As confirmed by the qualitative interviews, most participants (12/14) had negative experiences using the application when recording diet and discontinued its usage after a few days. It seemed that the application’s diet tracking component did not help them in managing GDM. Participants experienced difficulties with the search feature (3/12), measurement unit (8/12), and limited ethnic food database (9/12). For instance, the search feature did not bring up the commonly consumed items, and the everyday Chinese food items such as chicken rice were missing in the database:

*“[…] the food options are not very localized. The common local food, like chicken rice, cannot be found.” (P08)*

Another challenge was that the food item’s name was not worded in the commonly known way (1/12), and the imperial measurement (cup) in the application was not familiar to the participants. Instead, participants suggested to either allow them to upload pictures of their diet or to replace the measurement unit with “tablespoon” or “bowl”, which is the terminology commonly used by the dieticians who conduct the usual care education sessions. These participants (12/14) resorted to the usual procedure of a paper diary due to its ease of recording and convenience of viewing diet and blood glucose values side-by-side. 

Healthcare professionals tended to favor the paper diary because they had to navigate to different screens on the mHealth application to view the corresponding diet consumed for a particular blood glucose reading rather than being displayed on a single screen, as a paper diary does. Due to this, participants (12/14) perceived that their healthcare professionals were more interested in looking at the paper diary, which further discouraged them from using the application for data entry.

##### Reasons How Diet Tracking Component Was Useful

Despite the above-mentioned challenges, a few participants (2/14) used the application more frequently due to their perceived responsibility to the research team. They coped with the database’s limitations either by breaking down the ingredients or by choosing the most similar food type available in the application’s database. Additionally, they also mentioned that although they were not able to capture the precise food eaten, tracking diet using the application gave them a sense of control (2/2) and confidence in their food choices (2/2). By cross-checking the food item when their blood glucose readings were high, they were able to avoid the food item in future meals.

#### 3.1.3. Weight Tracking Behavior with Habits-GDM Application

Users were asked to monitor their weight once a week. The average number of weight values logged per week among the application users was 1.85 (SD = 1.60), with 116/170 users (68%) logging weight at least once a week.

##### Reason Why Weight Tracking Component Was Useful

Similarly, most interview participants (9/14) reported tracking weight using the application. Ease of use due to the automatic transfer of weight values from the digital weighing scale to the mobile phone (9/9) and GDM-related perinatal complications were the reasons reported for tracking weight. The graphical representation of the weight values recorded enabled straightforward interpretation and helped participants to track their progress over time (7/9).

##### Reasons How Weight Tracking Component Was Useful

By looking at the graphical representation of weight values, participants (7/9) felt they were on the right track in managing GDM because by looking at the weight values they could estimate if they could continue to eat in the same pattern or not. This type of self-monitoring increased their awareness. Five out of the fourteen participants interviewed did not use the application to monitor weight. Of these five participants, two mentioned monitoring their weight during the consultation as a reason for not using the application to record their weight. Three out of these five said that they did not perceive monitoring weight as a health priority during pregnancy. 

The graphical representation increased their self-awareness when managing GDM because with the graphical format, they could see how well they were managing their weight values over time and they could also review the data which was entered retrospectively. One participant viewed otherwise, perceiving the graphical representation of weight to induce weight-related anxiety, causing women to restrict dietary intake unnecessarily.

#### 3.1.4. Use of Coach Component of Habits-GDM Application 

Of all the coach messages, 162/189 (86%) were replies typed by the research coordinator in response to the logistics issues (i.e., a request for blood glucose testing strips and appointment confirmation) raised by the users. A minority (13/189, 7%) of these messages typed by the research coordinator were replies to the dietary clarifications raised by the users. The remaining messages were system update messages. 

##### Reasons for Using and Not Using the Coach Component

Consistent with these findings, many qualitative interview participants (10/14) reported sending messages to the coach via the chat interface only when they faced logistic issues. Participants (4/14) reported not using the coach component to ask queries about diet and glucose monitoring. The option of emailing healthcare professionals (2/4) and healthcare professionals in charge of their care not having direct access to the dashboard messages were the reasons (2/4). 

##### Reasons How Coach Component Was Useful

The automatic messages in response to high blood glucose readings were non-judgmental and encouraged some participants (6/14) to manage their GDM. Participants attributed the automatic messages to creating an immediate sense of self-awareness (5/6) in their food choices and motivated them for behavior change. However, they (5/6) thought its usefulness was only temporary because the messages delivered were always the same. They suggested having “personalized” and specific messages. For example, they preferred the messages to highlight “four carbohydrate portions were taken instead of three”. On the contrary, a few others (2/14) who had to use insulin to manage their GDM did not like the idea of taking suggestions from an application. For example, when they failed to achieve their target, having the application continue to give negative feedback demotivated them:

*“The messages are always replayed and standard. Always they will say you have to lower your carbs. After a while, you get used to the messages.” (P07)*

## 4. Discussion

The usage frequency of the application’s components varied greatly among the users. Tracking diet was the least commonly used component. The low usage was attributed to incorrectly worded food items and limited food database. However, a few interview participants mentioned perceiving a sense of control and confidence in their food choices when logging food using the application. On the contrary, the weight tracking component was the most used due to its ease of use. The automatic coach messages created an immediate sense of self-awareness in food choices and motivated behavior change according to some interview participants.

To concretize the key takeaways of this paper, the key results are mapped to the constructs of the Health Belief Model, and we provide suggestions for improvements to enhance application usage (Table 3 below). Briefly, our results fit into the constructs perceived benefit, barriers, self-efficacy, and cues to action. 

Educational lessons in the mHealth application have a key advantage as they provide vast quantities of health-related information which patients can frequently access at their convenience [42]. However, only 50% of the participants in the intervention arm of the SMART-GDM accessed the lessons. Our qualitative data showed that the easy availability of health-related information from the internet is a reason why the educational lessons in the application were not found to be useful by a few participants. Participants mostly accessed the internet for information soon after the diagnosis and while waiting for the hospital-organized education session. This highlights the importance of timing of mHealth interventions. Provision of the Habits-GDM application shortly after the diagnosis might have encouraged the utilization of educational lessons, reduced the known risk of accessing inaccurate health-related information from the internet [43], helped patients to prepare better to manage GDM, and enhanced the effectiveness of face-to-face sessions. 

Diet data entry was a key topic that affected the usability of the diet tracking component. Our findings showed that participants preferred to record the precise food eaten [44]. However, the application’s coaching system was designed using the principles of ecological momentary interventions [3,21,22]. Therefore, instead of collecting dietary information accurately in the form of a food diary, participants were cued through automated messages. The automated messages prompted participants to record their diet in the preceding 2–4 h when their 2 h post-meal glucose readings were above target. This maximized ecological circumstances for real-time reflections and learning [3]. Conveying the actual intention of recording diet and providing specific directions for patients to follow may have improved the application’s usage. Singapore’s diverse food environment with influences from the Chinese, Malay, Indian, and Western cultures [45] and the development of the application without users’ input could be the reasons for a difference in the naming of the food items [3]. Photo recording of diet for data entry has been shown to be accurate and easy to use. However, a few studies have reported its low detection accuracy with Chinese and Malay food due to the different appearances and ingredients used in these cuisines [46,47]. Therefore, applications which can recognize food based on the ingredients are needed [48]. Such applications can reduce not only the users’ time but also their effort [48]. Nevertheless, developing an easy-to-use diet tracking application with accurate information for a multi-ethnic population in Singapore is challenging. 

mHealth coaching by a healthcare professional has been shown to improve blood glucose targets in type 2 diabetes mellitus patients, as evidenced in a recent study by Koot et al. [49]. However, many of our study participants did not use the chat interface because of healthcare professionals’ lack of access to the e-coaching component. The Habits-GDM application was deliberately designed to minimize the need for intensive human resource coaching because the automated interactive coaching was intended to promote self-awareness of lifestyle choices [3].

### Limitations and Strengths

Our participants were, on average, 1.5 years postpartum when participating in this study. They may or may not remember their experiences with the application. However, firstly, to reduce recall error, we walked through the Habits-GDM application in detail with all the participants before conducting the interview. Additionally, participants were also allowed sufficient time for adequate recall of their experiences. Secondly, our study shares views of individuals who were interested in participating in the study. Therefore, it is possible that the results may be biased toward those individuals who chose to participate. Thirdly, only one author coded the majority of the transcripts. Nevertheless, we used specific strategies to ensure rigor and trustworthiness of the study, as described in the Methods Section of this paper. Lastly, due to the nature of qualitative research methods, caution should be taken when extending the findings beyond the sample.

To our knowledge, this is the first time a usage study of a largely automated mHealth application for GDM has been conducted in the Asia-Pacific region. This study is valuable from a health systems perspective because firstly, at a micro-level, using a mHealth application to manage GDM has been shown to improve patients’ satisfaction with their care and reduce unnecessary workplace absenteeism and travel costs [50]. Moreover, at a meso-level, it has been shown to reduce costs due to the need for fewer face-to-face appointments [51]. Singapore could benefit from using a mHealth application to manage GDM because most women of the reproductive age group are employed [1], and the costs of providing traditional care for patients with GDM are high [3]. Understanding patients’ perceptions of using a mHealth application to manage GDM is the first step. Future studies on healthcare professionals’ attitudes towards using a mHealth application for managing GDM in the Singapore context are required.

## 5. Practical Implications of This Study

The practical implication of this study is to understand if an mHealth application for GDM management can be incorporated in a clinical setting. mHealth interventions are complex because they involve multiple components with different behavioral constructs [18,19]. Hence, it is hard to predict which component will have the maximum impact on changing the behavior [18,19]. Therefore, before incorporating an mHealth application in a clinical setting, it is important to understand how well the application is received by the target user group. This will allow application developers to better optimize the mHealth application. This can, in turn, smoothen its incorporation in a clinical setting. Moreover, an RCT can only tell us whether an intervention worked or not. It cannot tell us ‘why’ the intervention worked and ‘why’ the intervention did not work [52]. Understanding this is important to enhance clinical application of the mHealth application.

## 6. Conclusions

Our findings suggested that the automated coaching in the mHealth application is effective in promoting self-awareness on healthy lifestyle choices among women with GDM, while reducing the need for human resources. However, the implementation of a mHealth application for GDM management is far from ideal. The inadequacies in some components of the application’s design resulted in poor user experience and less than ideal user engagement. Dietary management is an important element in GDM management. From our study, we learned that users found it tedious to track their diet using the existing design of the application. The context relevance of the food database is also a critical component to ensure continuous usage. Future work on a similar application should anticipate these potential challenges and attempt to address them during the development phase.

## Figures and Tables

**Table 1:** Habits-GDM application components and tracking frequency.

Component	Description	Tracking Frequency
Educational lessons	A total of 12 educational lessons on GDM ^1^ and self-management were delivered via a virtual coach. This curriculum was similar to the in-person education provided by the hospital’s usual care. It also contained additional modules on gestational weight gain.	Complete one lesson (lasting about 5–10 min) per day
Self-monitoring of blood glucose	Blood glucose measurements obtained using the Aina Mini glucometer (a novel hardware sensor that can be plugged into any smartphone) were automatically transferred into participants’ Habits-GDM application accounts.	Seven times a day for 2–3 days a week
Physical activity tracking	The Habits-GDM application tracks the number of daily steps taken using the participants’ built-in phone pedometers.	Planned physical activity of 30 min per day
Diet tracking	The food database takes reference from the Singapore Health Promotion Board’s Energy and Nutrient Composition of Food. Total calories and carbohydrates are the only two variables provided for each food.	At least three meals and two days per week
Weight tracking	Bluetooth-enabled weighing scale readings were automatically transferred to the application. Weight values are represented in a graphical, chart, or report format on the phone, in comparison to the ideal weight for baseline body mass index.	At least once a week
Coaching	An interactive messaging platform where participants are free to pose questions to the healthcare team who will respond in no more than 24 h. The healthcare team did not proactively approach the participants. Additionally, all participants receive health coaching via generic automated text messages on tips towards healthy behavior beneficial for GDM management. The food database was designed drawing from principals of ecological momentary interventions [21,22]. When the participant’s 2 h post-meal glucose readings were >6.6 mmol/L, they were cued through automated messages to record their diet in the preceding 2–4 h, maximizing ecological circumstances for real-time reflections and learning.	No recommendation provided

^1^ GDM—gestational diabetes mellitus.

**Table 2:** Coding scheme with count and percentage (n (%)) of subthemes and codes.

Theme	Subtheme	Code
Use of educational lessons of Habits-GDM application	Reasons why educational lessons were useful (9/14, 64%) and less useful (5/14, 36%)	Pictorial representation (6/9, 67%)
		Short duration (6/9, 67%)
		Easy to understand content (6/9, 67%)
		All information in one place (3/9, 33%)
		Basic content (3/5, 60%)
		Already available information on website (2/5, 40%)
	Reasons how educational lessons were useful (7/14, 50%) and less useful (9/14, 64%)	Easy to remember healthy foods (7/7,1 00%)
		Guided to make healthy food choices (7/7, 100%)
		Tiredness during pregnancy (9/9, 100%)
Diet tracking behavior with Habits-GDM application	Reasons why diet tracking component was less useful (12/14, 86%)	Difficult search feature (3/12, 25%)
		Limited food database (9/12, 75%)
		Incomprehensible measurement unit (8/12, 67%)
		Incorrectly worded food items (1/12, 8%)
		Healthcare professionals’ favor for paper diary (12/12, 100%)
	Reasons how diet tracking component was useful (2/14, 14%)	Sense of self control (2/2, 100%)
		Sense of confidence (2/2, 100%)
Weight tracking behavior with Habits-GDM application	Reasons why weight tracking component was useful (9/14, 64%)	Ease of use (9/9, 100%)
		Graphical representation (7/9, 78%)
	Reasons how weight tracking component was useful (9/14, 64%)	Increased self-awareness (7/9, 78%)
Use of coach component of Habits-GDM application	Reasons for using (10/14, 71%) and not using the coach component (4/14, 29%)	Logistic issues (10/10, 100%)
		Alternate modes to contact healthcare professionals (2/4, 50%)
		Healthcare professionals’ lack of direct access to dashboard (2/4, 50%)
	Reasons how coach component was useful (6/14, 43%)	Immediate sense of self-awareness in food choices (5/6, 83%)
		Usefulness temporary due to same messages (5/6, 83%)

**Table 3:** Themes, theoretical constructs, and suggestions for improvement.

Themes	User Perception	Construct	Suggestion for Potential Improvement to Enhance Application Usage
Use of educational lessons	All information in one place facilitated GDM ^1^ control	Perceived benefit	Increase convenience to access anytime
Diet tracking behavior	Low ease of use hindered tracking diet	Perceived barrier	Increase robustness of application component by incorporating local food with the commonly used local name
	Tracking generated confidence in food choices	Self-efficacy	To provide side-by-side display of diet data and blood glucose levels for patients to correlate
Weight tracking behavior	Weight, not a priority, hindered tracking weight	Perceived benefit	Enhance focus on the benefit of recommended gestational weight gain to reduce the risk of perinatal morbidity
	Weight monitored at consultation hindered tracking weight	Cues to action	Application to provide suggestions and cues to specific actions if patients are going off track and healthcare professionals to use and rely on application’s data
	Risk to baby facilitated tracking weight	Perceived benefit	Enhance focus on the benefit of recommended gestational weight gain to reduce the risk of perinatal morbidity
	High ease of use facilitated tracking weight	Perceived benefit	Increase robustness of application component
Use of coach component	Automated messages created an immediate sense of self-awareness in food choices	Self-efficacy	Increase robustness of application component
	Repetitive automated message content’s usefulness was temporary	Perceived benefit	Specific messages with specific actions when patients go off track or vary the language of the same message so that it is not too ‘automated’
	Healthcare professionals’ lack of access to dashboard prevented users from sending messages	Perceived barrier	Healthcare professionals to have access to the application and provide coaching
	Messages considered judgmental prevented users from sending messages	Self-efficacy Cues to action	Specific messages with specific actions when patients go off track and build specific cues to replace foods that are associated with high glucose to those with low glucose

^1^ GDM—gestational diabetes mellitus.

## Data Availability

Data will not be shared to protect the anonymity of the participants. Readers who wish to gain access to the data can write to the corresponding author, and data may be granted upon reasonable request.

## Supplementary Materials

The following are available online at https://www.mdpi.com/article/10.3390/ijerph18126670/s1, Figure S1: Screenshot of educational lessons, Figure S2: Tracking interfaces, supplementary materials 2: Interview guide, Table S1: Demographic characteristics and health information of participants, Table S2: Educational lessons’ usage.
