# Supplementary material for: Women’s Usage Behavior and Perceived Usefulness with Using a Mobile Health Application for Gestational Diabetes Mellitus: Mixed-Methods Study
Source: Int J Environ Res Public Health. 2021 Jun 21;18(12):6670. doi: 10.3390/ijerph18126670 (PMC8296439; doi:10.3390/ijerph18126670)
Supplement: Supplementary file 1 [file ijerph-18-06670-s001.zip › Supplementary material 2.pdf]

Supplementary material 2: Interview guide

Understand you were using the Habits App, let us walk you through the app for recall before we proceed.

a. Let us now talk about the educational lessons.
Qn1: Can you explain to us the educational lesson/s you found the most useful? (preferably 2 most useful)
Qn2: Can you share with us examples how this lesson has helped to manage your GDM?
Qn3: Can you explain to us the educational lesson/s which were the least useful? (preferably 2 least useful)
Qn4: Can you share with us examples how this lesson was not helpful?

b. Now regarding tracking your weight,
Qn1: Did you use this app to track your weight? (If No, Qn2) (If Yes, Qn3,5)
Qn2: Can you explain the reasons?
Qn3: Did tracking your weight with this app helped to manage your weight? (If No, Qn4) (If Yes, Qn5)
Qn4: Can you give us examples how it has not helped to manage your weight?
Qn5: Can you give us examples how it has helped?

c. I understand this App has a function where you can send messages to the coach,
Qn1: Did you send messages to the coach?
Qn2: The coach sends automatic messages based on your glucose levels. How was the experience with this function?
Qn3: Can you give us examples how interacting with coach has helped to manage GDM?
Qn4: Can you give us examples how it has not helped?

d. I understand this App has a function where you can enter diet data and get information on calorie and carbohydrate content,
Qn1: How was the experience with this function?
Qn2: Can you give us examples how interacting with this diet tracking function has helped to manage GDM?
Qn3: Can you give us examples how it has not helped?

Experiences with HCPs when using the app
Qn1: Can you share with me your experiences with HCPs when using this app? (prompts- did they review tracking from the app)

Thank you for your participation. We have come to an end of our interview.
