# Supplementary material for: Women’s Usage Behavior and Perceived Usefulness with Using a Mobile Health Application for Gestational Diabetes Mellitus: Mixed-Methods Study
Source: Int J Environ Res Public Health. 2021 Jun 21;18(12):6670. doi: 10.3390/ijerph18126670 (PMC8296439; doi:10.3390/ijerph18126670)
Supplement: Supplementary file 1 [file ijerph-18-06670-s001.zip › Supplementary Material 3 Tables.pdf]

Supplementary Material Table S1: Demographic characteristics and health information of the participants

Variable		RCT intervention group participants (*N* = 170)	Interview participants (*N* = 14)
Age (years), range (average)		24 – 43 (32)	27 – 36 (32)
**Ethnicity, n (%)**			
	Chinese	75 (44)	8 (57)
	Non-Chinese	95 (56)	6 (43)
Gestation at diagnosis of GDM, weeks (SD)		25.5 (3.2)	24.9 (4.1)
Gestation at delivery, weeks[a] (SD)		38.5 (1.9)	39.03 (1.1)
Family history of diabetes, n (%)		69 (42)	1 (7)
Insulin to manage GDM[b], n (%)		17 (10)	2 (14)

[a]Value for Gestation at delivery was calculated for 168 individuals since data was not available for two participants who delivered outside Singapore.
[b]GDM: gestational diabetes mellitus.

Supplementary Material Table S2: Educational lessons usage (N=84)

Lesson	Participants who accessed any lesson at least once, n (%)	Times each lesson was accessed, n (%)
Total	84 (100)	787 (100)
Glucose monitoring	56 (67)	127 (16)
Healthy eating	56 (67)	78 (10)
Introduction to Habits GDM[a]	49 (58)	72 (9)
Carbohydrates	48 (57)	67 (9)
Weight gain	47 (56)	65 (8)
Why treat	47 (56)	61 (8)
Eating smart	42 (50)	61 (8)
Understanding GDM[a]	44 (52)	56 (7)
Managing stress	43 (51)	55 (7)
Eating out	42 (50)	52 (7)
Looking ahead	35 (42)	47 (6)
Why exercise	38 (45)	46 (6)

[a]GDM, gestational diabetes mellitus.
