# Supplementary figures and images for: Women’s Usage Behavior and Perceived Usefulness with Using a Mobile Health Application for Gestational Diabetes Mellitus: Mixed-Methods Study
Source: Int J Environ Res Public Health. 2021 Jun 21;18(12):6670. doi: 10.3390/ijerph18126670 (PMC8296439; doi:10.3390/ijerph18126670)

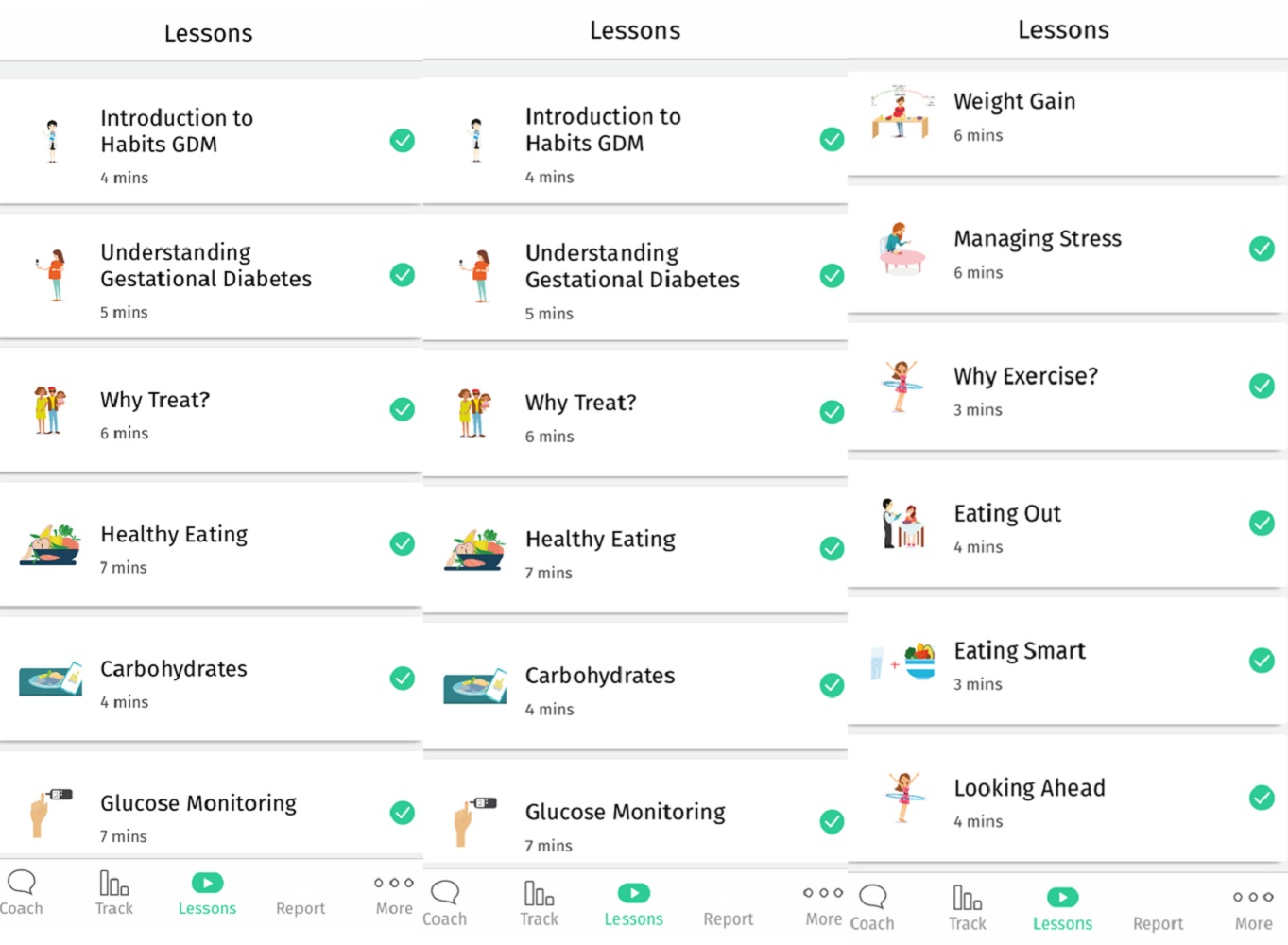

Supplement: Supplementary file 1 [file ijerph-18-06670-s001.zip › Figure S1 Screenshot of educational lessons of Habits-GDM application.jpg]

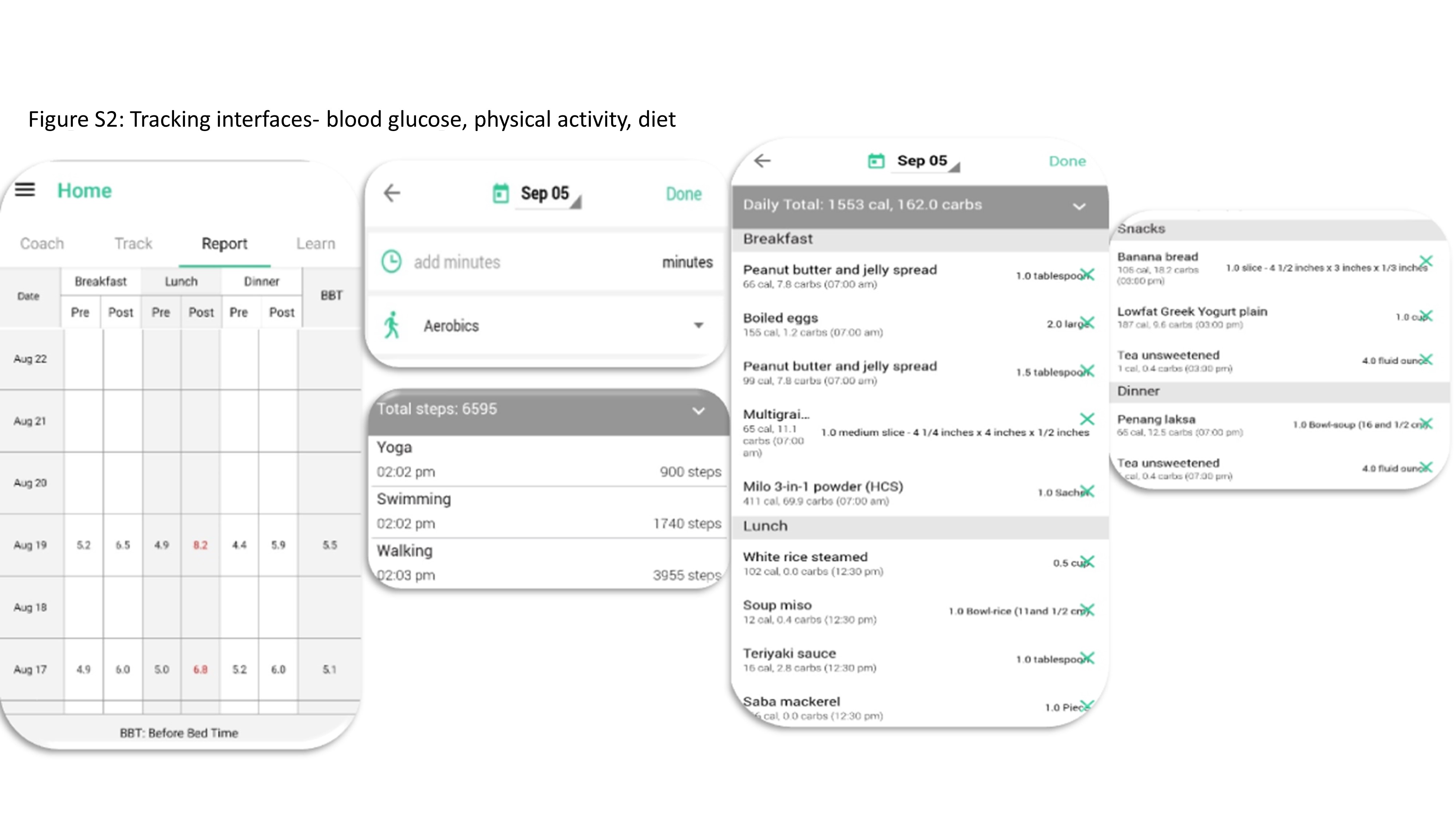

Supplement: Supplementary file 1 [file ijerph-18-06670-s001.zip › Figure S2 Tracking interfaces.jpg]
